# *In Vitro* Generation of Monocyte-Derived Macrophages under Serum-Free Conditions Improves Their Tumor Promoting Functions

**DOI:** 10.1371/journal.pone.0042656

**Published:** 2012-08-06

**Authors:** Flora Rey-Giraud, Mathias Hafner, Carola H. Ries

**Affiliations:** 1 Pharma Research and Early Development, Roche Diagnostics GmbH, Penzberg, Germany; 2 Institute of Molecular and Cell Biology, Mannheim University of Applied Sciences, Mannheim, Germany; University of Pittsburgh, United States of America

## Abstract

The tumor promoting role of M2 macrophages has been described in *in vivo* models and the presence of macrophages in certain tumor types has been linked to a poor clinical outcome. In light of burgeoning activities to clinically develop new therapies targeting tumor-associated macrophages (TAMs), reliable *in vitro* models faithfully mimicking the tumor promoting functions of TAMs are required. Generation and activation of human monocyte-derived macrophages (MDM) *in vitro*, described as M1 or M2 macrophages attributed with tumoricidal or tumor-promoting functions, respectively, has been widely reported using mainly serum containing culture methods. In this study, we compared the properties of macrophages originating from monocytes cultured either in media containing serum together with M-CSF for M2 and GM-CSF for M1 macrophages or in serum-free media supplemented with M-CSF or GM-CSF and cytokines such as IL-4, IL-10 to induce activated M2 or LPS together with IFN-γ to generate activated M1 phenotype. We observed differences in cell morphology as well as increased surface receptor expression levels in serum-containing culture whereas similar or higher cytokine production levels were detected under serum-free culture conditions. More importantly, MDM differentiated under serum-free conditions displayed enhanced tumoricidal activity for M1 and tumor promoting property for M2 macrophages in contrast to MDM differentiated in the presence of serum. Moreover, evaluation of MDM phagocytic activity in serum free condition resulted in greater phagocytic properties of M2 compared to M1. Our data therefore confirm the tumor promoting properties of M2 macrophages *in vitro* and encourage the targeting of TAMs for cancer therapy.

## Introduction

Macrophages derive from two different pools of circulating inflammatory or resident monocytes recruited to tissues in response to inflammation or infection as well as homeostasis to replace apoptotic resident macrophages [1], [2]. Several studies have also reported their involvement in tumorigenesis; the number of tumor-associated macrophages (TAMs) correlates with poor prognosis in breast, ovarian and prostate cancers [3]. The role of macrophages in anti- and pro-inflammatory immune processes results from their great heterogeneity and plasticity. Indeed, their phenotype is highly dependent on the specific microenvironment cues. By analogy to T helper cell (Th) nomenclature, macrophages have been classified into two categories: type 1 or classically activated (M1), and type 2 or alternatively activated (M2) [4], [5]. *In vitro*, M1 originate from monocyte stimulated with GM-CSF or M-CSF in the presence of IFN-γ and/or bacterial products such as LPS [6]. They are characterized by a pro-inflammatory phenotype, display microbicidal activity which results in tumor suppression, whereas M2 macrophages can promote tissue repair, matrix remodelling and angiogenesis supporting tumorigenesis. Three subtypes of M2 have been described *in vitro* according to the stimuli received. M2a, M2b and M2c are induced by M-CSF and IL-4 or IL-13, immune complexes together with LPS or IL-1β, and M-CSF and IL-10, respectively [4].

Changes in microenvironment signals *in vitro* lead to a switch of phenotype from M1 to M2 and vice versa [7], [8]. Moreover, under chronic inflammatory conditions, modification in macrophage phenotype *in vivo* has been suggested as a way for tumors to escape immune surveillance [9]. During early stage of tumor development, monocytes recruited to the tumor site encounter pro-inflammatory signals which contribute to their differentiation into M1 macrophages. In later stage of neoplastic transformation, signals from the tumor itself and from stroma cells promote changes towards an M2 macrophage phenotype, supporting further tumor progression [10].

Accessibility to patient tumor derived macrophages is limited and *in vitro* differentiation protocols faithfully recapitulating their activity are needed to test new therapeutic intervention strategies targeting specifically tumor-associated macrophages. However, previously published culture conditions of monocytes are highly variable and therefore might result in the generation of distinct MDM cell populations, due to their high plasticity. In order to confirm this hypothesis, we investigated the effect of two distinct culture media on the phenotype and more importantly the tumor promoting activity of MDM. Most reported protocols use stimulation with GM-CSF or M-CSF in media containing 10% FBS to generate M1 and M2, respectively [11]–[15]. However, since the exact composition of FBS is unknown and can differ from one provider to the other or from lot to lot, we used XVivo 10, media specifically developed for hematopoietic cells, supplemented with the cytokines mentioned above. We assessed the differences in morphology, surface receptor and cytokine expression as well as phagocytic activity and effect of MDM supernatant on tumor cell proliferation in these two culture conditions.

## Materials and Methods

### Cells and reagents

HCC1143 and T47D tumor cell lines purchased from ATCC and MOLT-4 line purchased from NCI were maintained in RPMI, 10% FBS, 2 mM L-Glutamine.

All cytokines were purchased from Biomol GmbH and LPS from Imgenex. Mouse anti-human antibodies were purchased from BD Pharmingen™: FITC-conjugated CD45 (clone 2D1), CD16 (clone 3G8), CD206 (clone 19.2), HLA-ABC (clone G46-2.6), CD64 (clone 10.1), CD86 (clone 2331); APC-conjugated CD14 (clone M5E2), CD71 (clone M-A712), HLA-DR (clone G46-6), CD1a (clone HI149) and CD11b (clone 44); PE-conjugated CD163 (clone GHI/61), CD23 (clone M-L233), CD32 (clone 3D3), CD80 (clone L307.4), CD68 (clone Y1/82A), and isotype controls FITC mouse IgG1 (clone MOPC-21), PE mouse IgG1 (clone MOPC-21), PE mouse IgG2b (clone 27–35), APC Mouse IgG2a (clone G155–178), APC Mouse IgG1 (clone MOPC-21).

The neutralizing TNF-α antibody was obtained from R&D Systems.

### In vitro generation of monocyte-derived macrophages (MDM)

The study was approved by the local ethics committee (Bayerische Landesärztekammer, Munich) and subjects gave written, informed consent. Monocytes were enriched from whole blood by negative selection using the Rosette Sep® monocyte enrichment cocktail (STEMCELL Technologies) according to the manufacturer's instructions. Briefly, blood from healthy donors was collected in Falcon tubes containing EDTA at 2 mM final concentration and incubated with enrichment antibody cocktail (50 µl per ml of whole blood) at room temperature for 20 minutes. Cells were then separated by density gradient using Ficoll-Paque™ PLUS (GE Healthcare). Platelets present in the enriched monocyte fraction were discarded by 3 washing steps in PBS, 2% FBS. Finally, monocytes were seeded in either XVivo 10 (Cambrex) or RPMI 10% FBS, 4 mM L-Glutamine with Pen/Strep at a concentration of 5×10^5^ cells/ml in 12-well tissue culture treated plates for 6 days in the presence of either 100 ng/ml rHuGM-CSF (M1) or 100 ng/ml rHuM-CSF (M2). For M2a and M2c polarization, monocytes were incubated in XVivo 10 with rHuM-CSF and 10 ng/ml rHuIL-4 or 10 ng/ml rHuIL-10, respectively. For M1 activation, monocytes were first incubated with rHuM-CSF or rHuGM-CSF for 3 days followed by stimulation with 10 ng/ml LPS and 50 ng/ml rHuIFN-γ for 3 additional days. As indicated by the manufacturer, XVivo 10 media contains human albumin, recombinant human insulin, and human transferrin. It does not contain any exogenous growth factors, artificial stimulators of cellular proliferation, undefined supplements, or protein kinase C stimulators.

Changes in cell morphology were assessed by phase contrast microscopy (Axiovert 135, Zeiss). Phenotypical and functional characterization of MDM was performed after 6 days.

### Flow cytometry

MDM were detached from the plates by incubation at 37°C with PBS containing 5 mM EDTA. Cells were washed once in PBS, 5% FBS at 4°C, blocked for 30 minutes on ice with human IgG (Invitrogen™) and stained for 1 h on ice with conjugated antibodies or matching isotype controls as stated before. Intracellular expression of CD68 was assessed using BD™ Phosflow Lyse/Fix Buffer and BD™ Phosflow Perm Buffer II as instructed by the manufacturer. DAPI staining was performed to discriminate dead from live cells.

Sample acquisition was performed using a FACSCanto™ II (BD Biosciences) and geometric mean fluorescence intensities (MFI) were analyzed in FlowJo 7.5.5 software (Tree Star, Inc).

### Multiple analyte profile (MAP) for cytokine level determination from MDM supernatant

Media from 6-day culture of MDM were harvested, centrifuged and stored at −20°C. For analysis, samples were thawed and kept at room temperature.

Expression level of 42 cytokines was determined using MILLIPLEX® MAP Human Cytokine/Chemokine Panel (Millipore™) according to the manufacturer's instructions. Samples were run in duplicate. Briefly, standard, quality controls and samples were added to a 96-well filter plate together with capture antibodies coated-beads. Plate was incubated overnight at 4°C under agitation. Unbound beads were washed away and detection was performed by addition of bead-specific biotinylated detection antibodies and streptavidin-PE conjugate. Fluorescence signal was determined on Luminex® 100/200™ System and data were analyzed using Excel software.

### Human tumor cell proliferation assay

Tumor cells were seeded in 96-well plates, at 5×10^3^ cells/ml. MDM conditioned media (CM) or control media (XVivo 10 or RPMI 10% FBS) were used to culture tumor cells for 5 days at 37°C, 5% CO_2_ in the presence or the absence of 10 µg/ml anti-TNF-α. Cell proliferation was determined using CellTiter-Glo® Assay (Promega) as recommended by the manufacturer. Luminescence signal was recorded on an Infinite® 200 PRO (Tecan) and data were analyzed using Excel software. Data represent results from three independent experiments with each sample run in triplicate.

### Phagocytosis assay

MOLT-4 were maintained in RPMI, 10% FBS, 2 mM L-Glutamine and stained for functional assay with CellTracker™ Green CMFDA (Molecular Probes™) according to manufacturer's instructions. Cells were killed by three freeze-thaw cycles performed in liquid nitrogen and 1×10^5^ cells were added to monocyte-derived macrophages (activated M1, M2a and M2c) for 4 h at 37°C, 5% CO_2_. Non-ingested MOLT4 were removed, MDM detached from the plate using PBS containing 5 mM EDTA and stained for 30 min on ice with APC-conjugated CD11b antibody. Dead/live cells were discriminated by DAPI staining. Fluorescence measurements were performed on a FACSCanto™ II (BD Biosciences) and analyzed by FlowJo 7.5.5 software (Tree Star, Inc). Negative control corresponded to MDM incubated with MOLT4 in PBS and % phagocytosis represented the proportion of live MDM (DAPI^−^ CD11b^+^) that acquired green fluorescence (CMFDA^+^).

### Statistics

Data were presented as Mean ± SEM of n experiments as indicated in the figure legends. Statistical analysis was performed using the JMP software (version 8, SAS institute). Statistical significance between conditions was determined using pairwise t-tests (without multiple testing corrections) or pairwise comparison applying the Tukey-Kramer method. P values<0.05 indicate significance (*).

## Results

### Heterogeneity in MDM morphology

Monocytes were freshly isolated from whole blood from healthy donors using a two-step negative selection as described in Material and Methods. Enriched CD14^+^, CD16^+or−^ round-shaped monocytes were further cultured in the presence of GM-CSF or M-CSF for 6 days, in either RPMI 10% FBS or XVivo 10 media, respectively to allow differentiation of monocytes into M1 or M2 macrophages.

**Figure 1:**
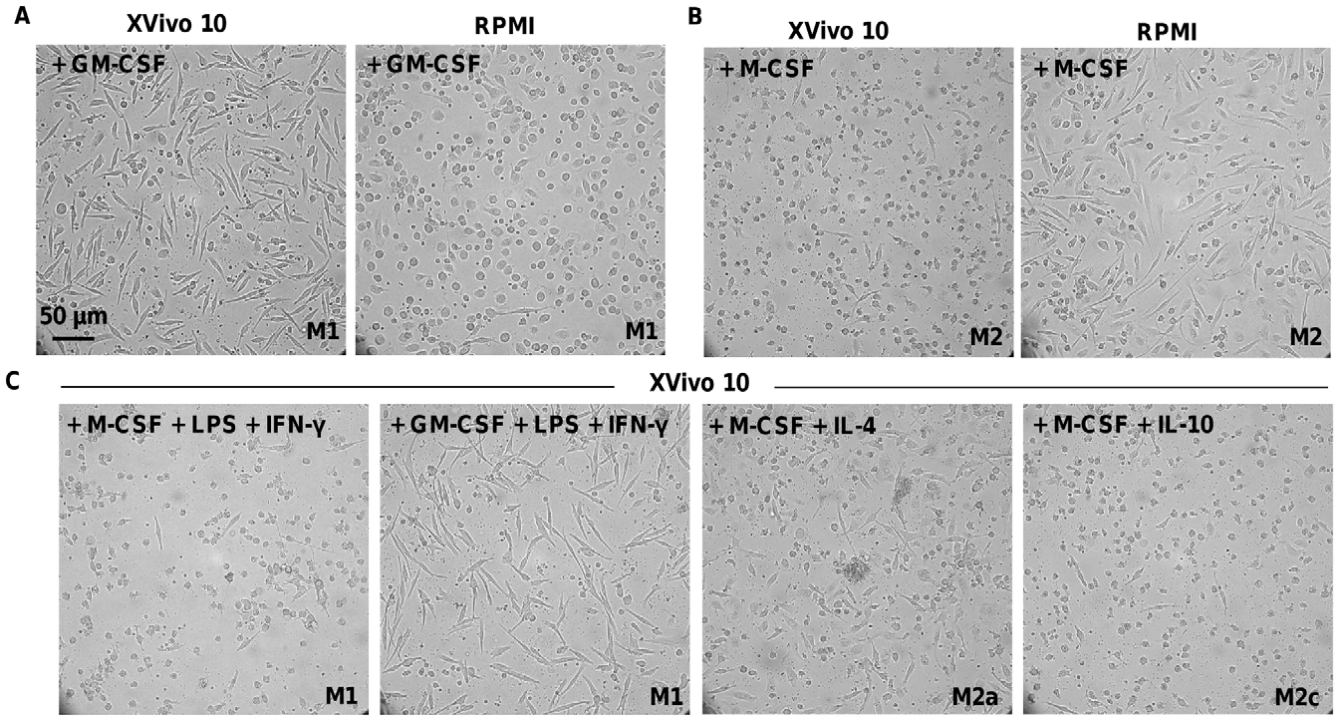
Morphology of monocyte-derived macrophages after 6 days. Differentiation in the presence of GM-CSF (**A**) or M-CSF (**B**) in Xvivo 10 or RPMI +10% FBS. (**C**) Differentiation in XVivo 10 media supplemented with the indicated cytokines, as described in Material and Methods. The scale denotes 50 µm.

Monocyte-derived macrophages presented a unique morphology dependent on both the culture media and the cytokine stimulation used. Stimulation with GM-CSF in XVivo 10 revealed a different morphology compared to RPMI 10% FBS which was also visible for M-CSF differentiated macrophages in serum-free and serum-containing media. GM-CSF in XVivo 10 or M-CSF in RPMI 10% FBS led to a majority of elongated, fibroblast-like shaped cells whereas the presence of M-CSF in XVivo 10 or GM-CSF in RPMI 10% FBS induced a majority of round or oval macrophages (**Figures 1A and 1B**). Similar tendency was observed among donors, albeit with variations in the proportion of round versus elongated cells from one donor to the other (data not shown).

In addition, enhanced adherence properties of elongated macrophages compared to round cells were observed. Indeed, MDM stimulated with GM-CSF in XVivo or M-CSF in RPMI required longer treatment with PBS containing 5 mM EDTA in order to detach from the culture plate in comparison to MDM stimulated with M-CSF in XVivo 10.

The addition of IL-4 or IL-10 to M-CSF – leading to differentiation into M2a or M2c MDM, respectively - did not result in changes in macrophage morphology (**Figure 1C**). However, stimulation by LPS and IFN-γ led to an increase in detachment of M1 MDM.

### M1 and M2 macrophage receptor expression is higher in RPMI 10% FBS compared to XVivo 10

Expression levels of 14 surface markers and 1 intracellular receptor were assessed by flow cytometry and compared to receptor expression on freshly isolated monocytes (**Table S1A**). Data represent the average level of receptor expression within a minimum of eight donors as indicated in Table S1.

**Figure 2:**
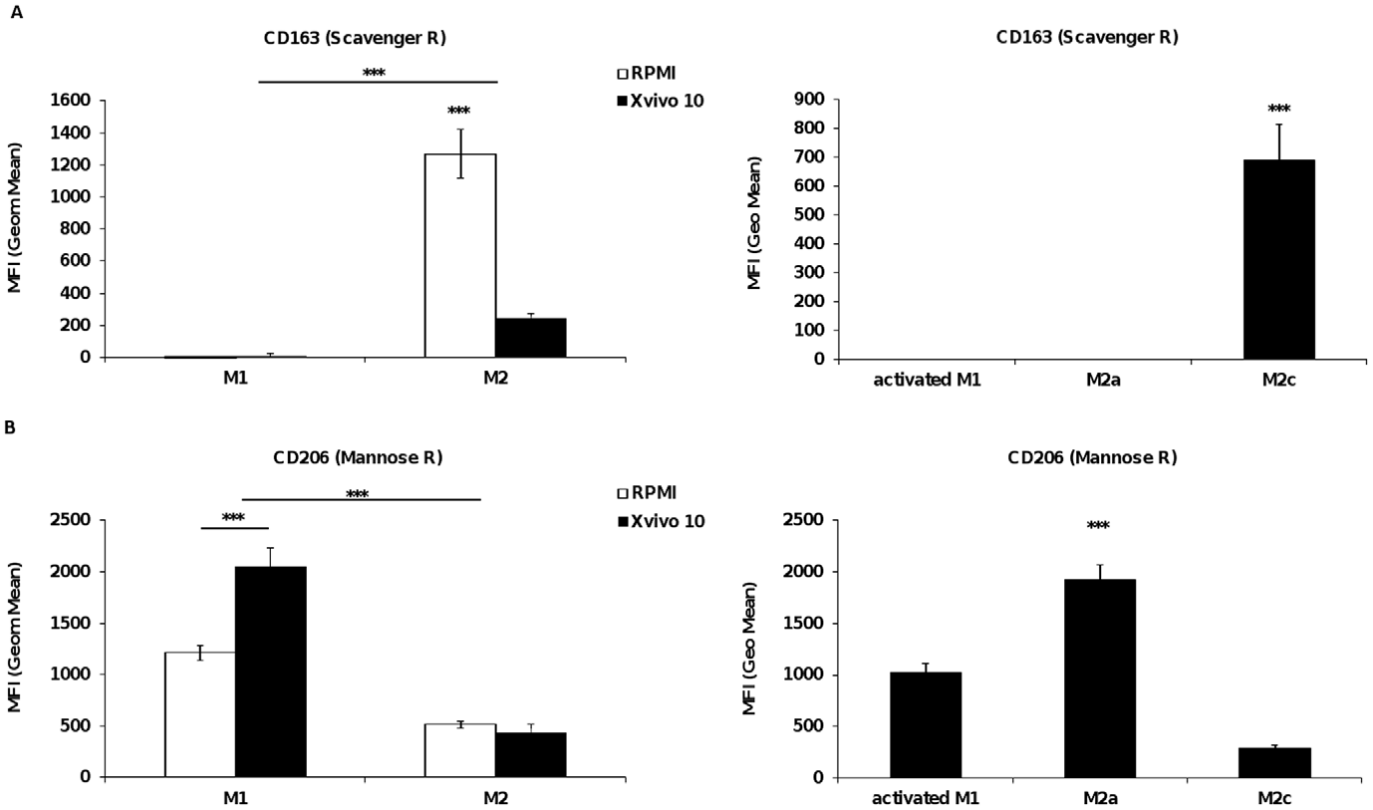
Receptor expression level on monocyte-derived macrophages. Surface expression of CD163 (A) or CD206 (B) on M1 and M2 MDM in Xvivo 10 or RPMI +10% FBS, or on M1, M2a and M2c MDM in XVivo 10 media. Data represent mean ± SEM of Mean Fluorescence Intensity (Geom. mean) of at least 8 donors. Statistical significance was determined using Tukey-Kramer HSD test pairwise comparison (***p<0.001).

When compared to expression level on monocytes, levels of CD45, CD68, CD71, CD64 (FcγRI), HLA-ABC (MHC class I) and HLA-DR (MHC class II), CD86 and CD206 (mannose receptor) were increased during differentiation into macrophages, independently of the culture conditions or macrophage subtype. Moreover, M1 and M2 shared similar expression level of MHC class I, FcγRI and CD86 receptors as well as intracellular CD68 (**Table S1A**). As previously reported, M1 were characterized by the expression of CD80 and the absence of CD163 (scavenger receptor, Figure 2A) compared to monocytes whereas M2 macrophages expressed higher level of CD14, CD163, CD32 (FcγRII) and CD16 (FcγRIII) than M1 macrophages and lack CD80 expression [4].

Differences in expression level were detected between RPMI 10% FBS and XVivo 10. Importantly, a subset of M1 macrophages from culture in serum-containing media expressed CD1a, a dendritic cell (DC) marker [11], [16], suggesting the presence of undefined soluble factors, such as IL-4, in RPMI or more likely FBS sustaining, together with GM-CSF, monocyte differentiation into a DC-like phenotype (**Figure 1A**). This observation correlates with both cell morphology and decrease in CD14 expression in serum-containing media; dendritic cell being round and lacking the receptor on their surface upon differentiation. In addition, CD80 and to a lesser extent CD45 were expressed at a significantly higher level in RPMI 10% FBS than in XVivo 10 whereas CD206 expression was higher in Xvivo than in RPMI 10% FBS (p values<0.05, Figure 2B). On the other hand, M2 macrophage markers CD14, CD32, CD16 and CD163 were significantly higher expressed in RPMI 10% FBS (p values<0.05) compared to XVivo 10. In both media, expression of CD80, CD23 (FcεRII) and CD1a was absent. Level of receptor expression in both M1 and M2 MDM appeared thus higher in the presence of serum than in serum-free conditions.

### Receptor expression on M2a and M2c subpopulations in comparison to M1 activated macrophages

MDM differentiation into M2a and M2c was performed in XVivo 10 by incubation with M-CSF and IL4 or IL-10, respectively. On the other hand, M-CSF or GM-CSF differentiated MDM were further activated with LPS and IFNγ for 72 h, leading to M1 activated macrophages. As reported previously [4], [7], [17], we also observed down-regulation of CD14 and CD163 surface expression on M2a (**Figure 2A** **and Table S1B**). Furthermore, this M2 cell subtype displayed increased expression of CD206 and CD23, a hallmark of M2a differentiation [18]. We also observed an increase in co-receptor CD86 expression on M2a. In contrast to IL-4, the addition of IL-10 led to higher expression of receptors detected on the M2 population such as CD14, FcγRI, FcγRII, and CD163 associated with low CD206 [19], [20], HLA-DR expression and the absence of CD23. In summary, the receptor repertoire expressed on M2c was identical to the M2 macrophages, yet with enhanced expression levels, whereas the M2a population differed from the M2 in the type of receptors expressed.

Activated M1 displayed increased CD80 surface expression (**Table S1B**). Surprisingly, we also observed marked differences in the M1 populations dependent on the presence of M-CSF compared to GM-CSF. CD14 and CD16 expression was significantly higher in LPS and IFN-γ activated M1 macrophages differentiated in the presence of M-CSF than in GM-CSF. This upregulation was also observed in M-CSF differentiated M2 macrophages, suggesting that these markers are regulated by M-CSF and can be further upregulated especially by LPS. In contrast to the LPS receptor CD14, costimulatory receptor CD86 or HLA-DR were higher expressed in GM-CSF dependent activated M1 macrophages, indicating a regulation of expression under the control of GM-CSF. Finally, CD71 expression was higher in M2a/M2c compared to M1 macrophages, correlating with data supporting a differential regulation mechanism of the transferrin receptor in M1 versus M2 [21], [22]. Receptor expression on activated M1, M2a or M2c in serum-free condition thus confirmed reported data on MDM phenotype.

### Differential cytokine secretion between XVivo 10 and RPMI 10% FBS culture conditions

**Figure 3:**
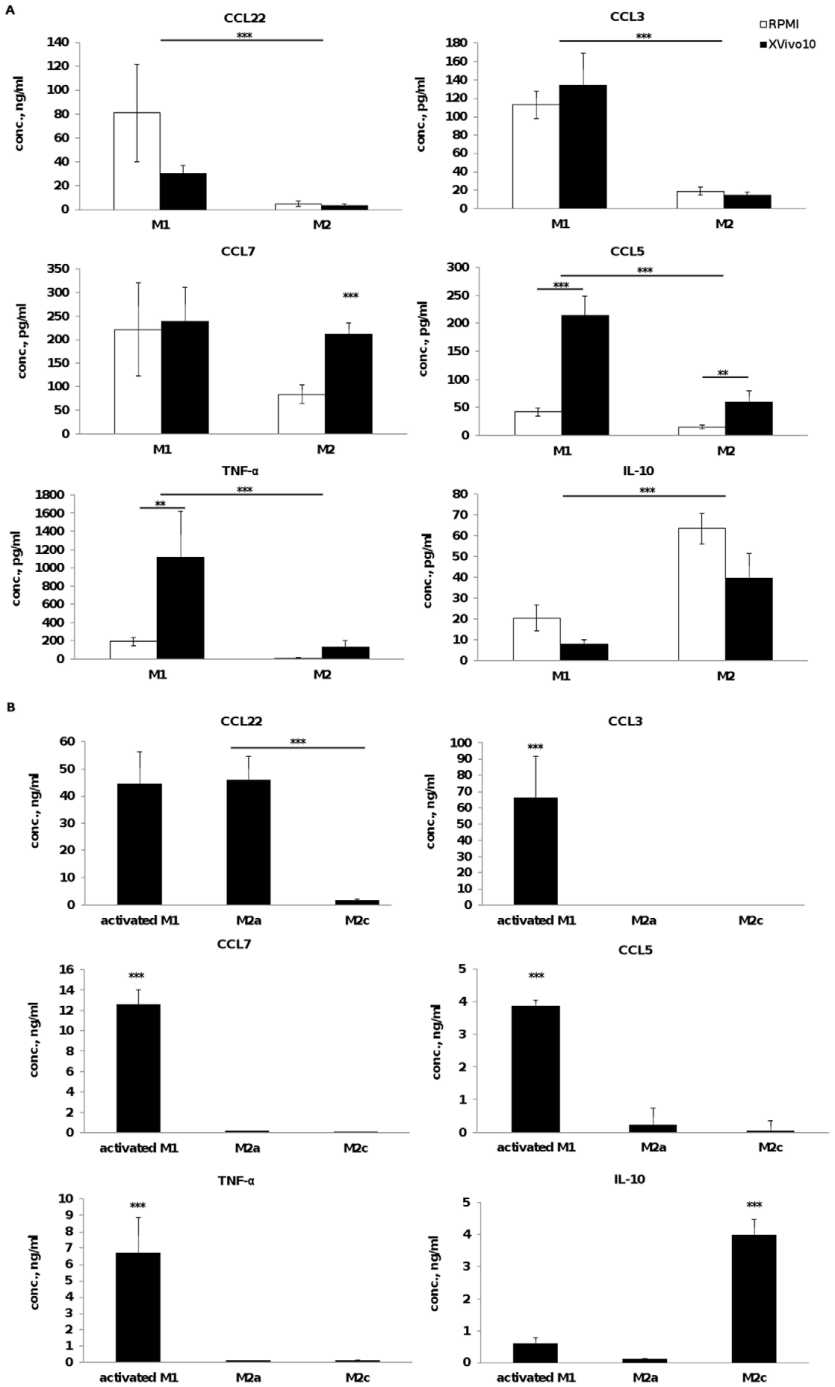
Cytokine concentration in supernatant of monocyte-derived macrophages. Monocytes were stimulated for 6 days with (**A**) GM-CSF (M1) or M-CSF (M2) in XVivo 10 or RPMI +10% FBS, or with (**B**) GM-CSF for 3 days and LPS and IFN-γ for 3 additional days (M1), M-CSF+IL-4 (M2a) or M-CSF+IL-10 (M2c) in XVivo 10. Data represent mean ± SEM of cytokine concentration of at least 8 donors. Statistical significance was determined using Tukey-Kramer HSD test pairwise comparison (** p<0.005, ***p<0.001).

Levels of 42 cytokines and chemokines in MDM supernatant were determined using the MAPx technology. Direct comparison of cytokine levels between MDM subtypes was conducted owing to a similar number of cells in culture upon differentiation, regardless of the stimulation applied (data not shown). Among the 42 cytokines measured, 30 could be detected above background expression level and 14 showed significant differences in expression levels between M1 and M2 MDM (**Figure 3** **and Table S2A**). However, a number of factors were secreted at extremely low level (<50 pg/ml). MDM released high levels of both chemokines CCL2 and IL-8, regardless of the stimulation applied. The levels of cytokine expression were highly variable among donors. However, compiling results from 8 distinct donors showed that levels of CCL3 and TNF-α were higher in M1 compared to M2 supernatant, correlating with the pro-inflammatory nature of these cytokines. On the other hand, the level of IL-10 was higher in M2 supernatant. In both macrophage subpopulations, PDGF-AA and Gro levels were higher in MDM supernatant cultured in XVivo 10 as compared to RPMI. Similarly, IL-1ra and CCL7 secreted by M2 as well as TNF-α, IL-1α, IL-1β, and CCL5 released by M1 were higher in XVivo 10 than in RPMI. In summary, cytokine levels detected in M1 or M2 CM appeared to be higher in serum-free compared to serum-containing media.

### Cytokine and chemokine profiles of activated M1 and M2a and M2c subpopulations

Levels of IL1ra [4], [23], CCL5 and CCL22 were increased in M2a whereas no effect on the level of these cytokines was detected in M2c compared to M-CSF alone. In addition, Gro levels decreased while EGF increased in M2a and M2c supernatants in comparison to M2 macrophages (**Figure 3** **, Table S2B**). On the other hand, pro-inflammatory cytokines such as IL-1α, IL-1β, IL-6, TNF-α, IL-12p70, CCL3 and CCL4 were upregulated in M1 MDM in the presence of LPS + IFN-γ (**Figure 3** **and Table S2B**) when compared to M2. Activated M1 macrophages displayed a different cytokine profile dependent on the presence of M-CSF and GM-CSF. M-CSF regulated M1 macrophages secreted higher IL-10 and VEGF combined with lower level of TNF-α compared to GM-CSF treated M1 macrophages, indicating a less pronounced M1 phenotype despite the presence of LPS, a strong inducer of the pro-inflammatory phenotype. Our data demonstrated increased production of specific cytokines by activated M1, M2a and M2c correlating with the presence of MDM phenotype induced cytokines, LPS+ IFN-γ, IL-4 and IL-10.

### Differential effect of MDM conditioned media on tumor cell proliferation

M1 and M2 macrophages were further characterized with regards to their influence on tumor cell proliferation. M1 and M2 conditioned media from 6-day culture in serum-containing or serum-free media were transferred to monolayers of the human breast tumor cell line HCC1143, and proliferation was determined after 5 days.

**Figure 4:**
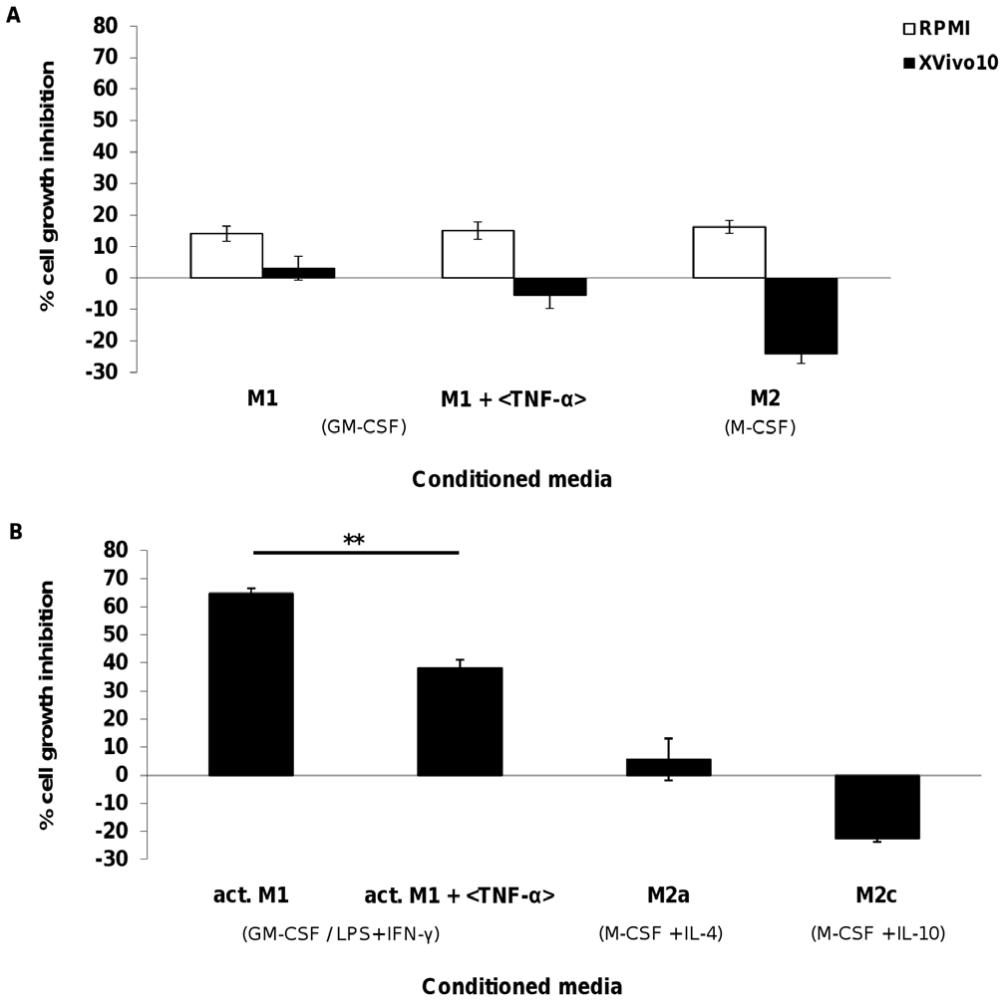
Effect of monocyte-derived macrophage supernatant on HCC1143 cell line proliferation. Evaluation was performed in the presence or the absence of neutralizing TNF-α antibodies, in (**A**) conditioned media from M1 or M2 MDM cultured in RPMI 10% FBS or XVivo 10, (**B**) conditioned media from activated M1 (act. M1), M2a and M2c MDM stimulated in XVivo 10. Identical y-axis scales were used for comparison sake. Data represent mean ± SEM of three independent experiments including each condition in triplicate. Statistical significance was determined using t-test pairwise comparison (** p<0.005).

Both M1 and M2 conditioned media from culture in RPMI 10% FBS induced tumor cell growth inhibition at a similar level. However, different results were obtained when using CM from XVivo 10 culture. Indeed, M1 CM slightly reduced cell proliferation whereas M2 CM increased HCC1143 proliferation by 25% (**Figure 4A**). Decrease in cell proliferation was TNF-α independent as shown by the absence of effect upon addition of a neutralizing TNF-α antibody to the culture. We then assessed the effect of CM from activated M1, M2a and M2c culture on the tumor line viability (**Figure 4B**). HCC1143 proliferation was not affected in the presence of M2a CM whereas M2c increased cell growth by 25%. In activated M1 CM, HCC1143 proliferation was reduced by 60%. Inhibition of proliferation could be partially rescued by a neutralizing TNF-α antibody. IFN-γ and TGF-β neutralizing antibodies had no effect on tumor cell inhibition (data not shown). The data presented here were also confirmed using T47D and other tumor cell lines (**Figure S1** and data not shown). Our data not only revealed significant differences in tumor cell line responses towards MDM between serum-containing and serum-free media, but also correlated with the pro- and anti-tumoral nature of M1 and M2 type macrophages, respectively. Moreover, serum-free condition culture allowed the identification of tumor cell lines responsive to M2 conditioned media such as HCC1143 or T47D.

### Phagocytic properties of activated M1 and M2 MDM in XVivo 10

**Figure 5:**
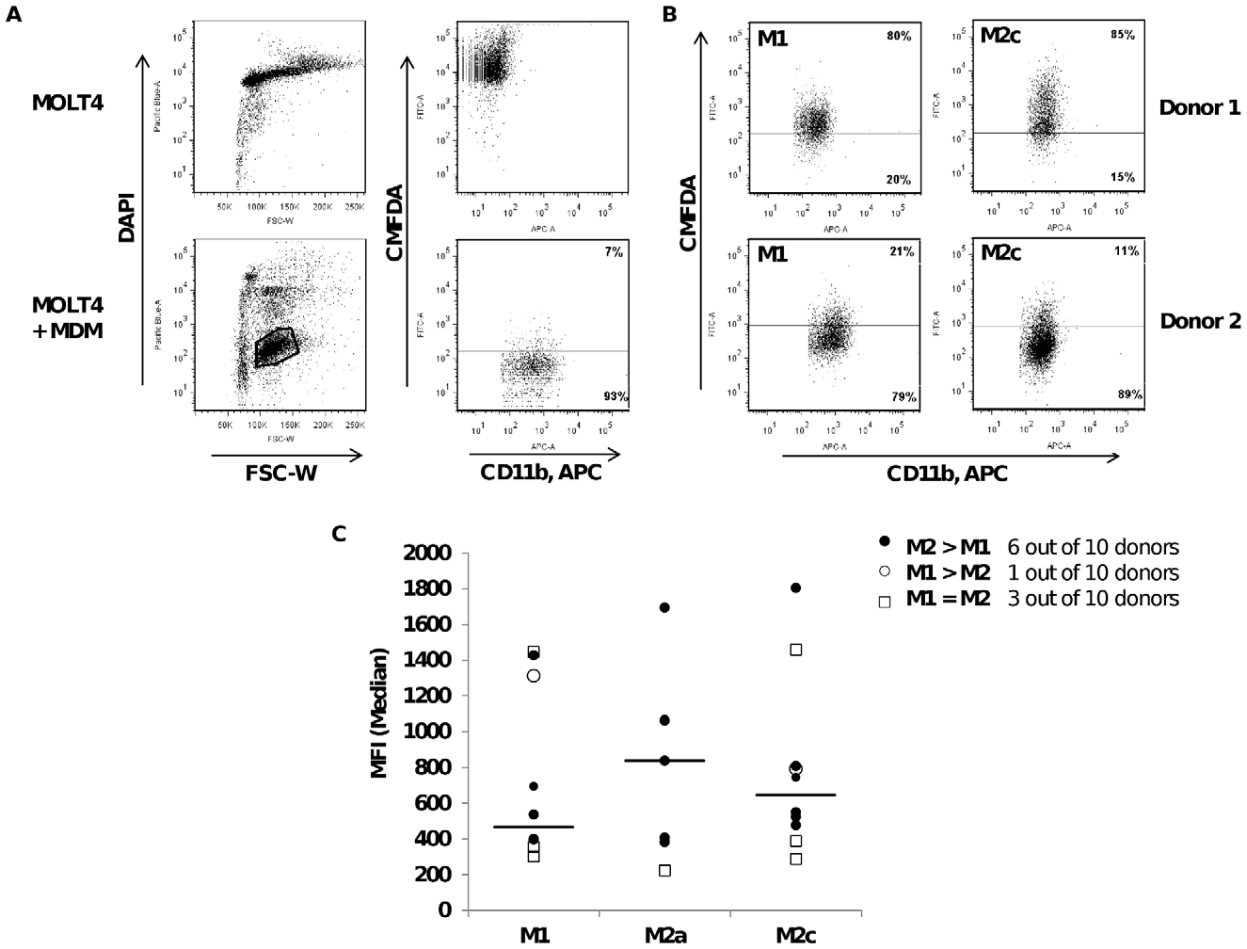
Phagocytic activity of M1, M2a and M2c monocyte-derived macrophages towards MOLT4. Gating strategy used to assess phagocytosis of MOLT4 debris by MDM obtained by flow cytometry: controls included assessment of MOLT4 alone or together with MDM in PBS (**A**). Dot plots representing data from two distinct donors in XVivo 10 as described in Methods (**B**). Mean fluorescence intensity (median) of phagocytosing MDM derived from ten distinct donors, attributed with identical (□) or differential (• or ○) activity between activated M1 and M2a or M2c MDM (**C**). Horizontal bars represent the median value of each group.

Since only the M1 and M2 MDM differentiated and activated in serum-free conditions reflected both MDM pro and anti-tumoral activity, we studied their phagocytic activity towards leukaemia cell line MOLT4 debris. Leukaemia cell lines have been used before to study antibody-independent MDM phagocytosis [24]. Target cells were stained with green membrane dye CMFDA, killed by repeated freeze/thaw cycles and incubated for 4 h together with MDM in XVivo 10 or PBS serving as a negative control. Uptake of target cells (DAPI^+^ CD11b^−^) by MDM was monitored by flow cytometry and gating was performed on live MDM (DAPI^−^ CD11b^+^), as represented in **Figure 5A**. The frequency of phagocytosed MOLT4 particles was defined by the proportion of CD11b^+^ MDM that acquired green fluorescence (**Figure 5B** **, upper quadrant**). Activated M1, M2a and M2c MDM from ten different donors were tested and the proportion of CD11b^+^ Green CMFDA^+^ effector cells represented 5 to 80% MDM regardless of the stimulation, indicating high variability in phagocytosis activity among donors. When analyzing the median fluorescence intensity of phagocytosing cells, MDM could be divided into three groups with regards to differences in activity between M1and M2a or M2c (**Figure 5C**). In the first one, M2a and M2c MDM derived from six donors, among which donor 1 (depicted in **Figure 5B**), exhibited greater phagocytic properties compared to activated M1 MDM. The second group consisted of one donor which activated M1 MDM showed higher phagocytic activity than M2a or M2c MDM whereas the third group included activated M1 and M2 MDM, derived from three donors, displaying identical functionality such as in donor 2. In summary, our data demonstrated that M2a and M2c MDM exhibited greater phagocytic activity compared to activated M1 MDM despite individual donor variability, correlating with the scavenging properties of M2 macrophages expressing higher level of surface receptors involved in debris clearance compared to M1 MDM.

## Discussion

Although various protocols for *in vitro* differentiation of macrophages have been published, a comparison and detailed characterization of macrophages generated in different culture conditions with regards to their tumor promoting function has to our knowledge not been described yet. Particularly with the advent of emerging therapeutic strategies aiming to target the tumor promoting functions of M2 macrophages, a robust and well defined assay capturing these macrophage properties is needed.

We demonstrated that MDM culture in serum-free media represents a more adequate model to study macrophages *in vitro* in comparison to serum-containing conditions. We found that albeit similar cell surface marker and cytokine repertoire, the cells exhibited great differences in terms of morphology and function.

We observed that M1 as well as M2 in serum-free condition displayed a different morphology compared to culture in serum-containing media. Unpredictable factors present in FBS could activate signaling pathways involved in cell cytoskeleton regulation, leading to modification in cell morphology. Furthermore, changes in cell adherence to plastic from one condition to the other suggested a differential regulation of surface adhesion molecules in the different culture condition tested.

Furthermore, MDM generated in serum containing or serum-free culture conditions displayed differential activity. Functional studies revealed opposite effects of macrophage derived CM on the proliferation of HCC1143 and T47D. Unlike data obtained in 10% FBS, results in serum-free conditions correlated with the anti- or pro-tumor properties of M1 or M2, respectively. However, no correlation between receptor expression and functional activity was detected implicating that not the expression level but rather the presence of CD163 is crucial. Indeed, CD163^+^ macrophages have been described as highly pro-tumoral and infiltration of CD163^+^ TAMs has been correlated with poor prognosis [25]–[27].

Following these observations, we focused on MDM originating in XVivo 10 and characterized M1, M2a and M2c MDM. Their phenotype correlated with previously published data [4], [18]. Even though the role of M-CSF in skewing macrophages towards the M2 phenotype is well documented, we tested if strong M1 inducing stimuli such as LPS were able to override the M-CSF mediated effects. Indeed, the tumoricidal effect of M1 conditioned media has been previously described [28]. Inflammatory cytokines were secreted in comparable levels by M-CSF as well as GM-CSF activated M1 MDM. However, M-CSF activated M1 macrophages displayed in parallel a more pronounced immuno-regulatory and angiogenic phenotype, implicating that both M1 and M2 signals are integrated by the macrophages, resulting in a mixed M1/M2 phenotype. This finding might explain why antibodies activating the anti-tumoral functions of macrophages such as the CD40 antibody, only transiently skew macrophages towards the M1 program in an immunosuppressive environment [29].

On the other hand, functional studies showed that M1 conditioned media inhibited cell proliferation in a TNF-α dependent manner [30]. This data were supported by the increased TNF-α level measured in M1 supernatant in the presence of LPS and IFN-γ. Additional soluble factors were involved in the process, as demonstrated by an only partial rescue of inhibition by neutralizing TNF-α antibody. Importantly, even though receptor expression was elevated on activated M1 in RPMI 10% FBS, lower TNF-α cytokine level in MDM supernatant was detected. Hence, the data presented in this study suggest that the cytokine profile but not the receptor repertoire of M1 macrophages correlates with their activity. This finding cautions to consider not only marker expression for characterization of M1 macrophages in tumor tissue but also their secreted cytokines. However, the detection of soluble factors in tissue is technically challenging.

As to M2 conditioned media, tumor cell response to the soluble factors secreted varied from one line to the other, suggesting that each tumor cell line responded differently to soluble factors contained in this conditioned media. We could here identify tumor cell lines which responded to M2 conditioned media under serum-free conditions only, suggesting that this particular milieu better mimics the situation *in situ* in opposition to serum-containing culture. The presence of growth factors such as EGF combined with the absence of pro-inflammatory cytokines in M2 supernatant, in contrast to M1, may promote cell proliferation. On the other hand, the HCC1143 cell line did not sustain monocyte survival (data not shown) most likely owing to a lack of growth factor release and correlating with studies that have demonstrated the role of stroma cells besides TAMs in shaping monocytes towards a pro-tumoral fate [31].

In order to confirm that serum-free culture conditions were suitable for macrophage functional studies *in vitro*, we assessed their phagocytic activity towards leukaemia cell line debris to better mimic the physiologic scavenging function [24]. Few data are available on the comparison of antibody-independent phagocytosis by M1 versus M2 macrophages. Indeed, phagocytic activity of MDM is generally assessed using beads, bacteria/yeast particles or tumor cells in the presence of an antibody [13], [32], [33]. Despite differences in phagocytosis activity among donors, we showed here that M2 displayed a greater activity towards tumor cell debris in comparison to activated M1, in the absence of opsonisation by antibodies.

Moreover, MDM generated in serum containing media with GM-CSF – M1 type - were characterized by the presence of a CD1a^+^ subpopulation. Thus, the so-called M1 macrophage population in serum-containing media comprised both macrophages and dendritic cells. This heterogeneity in cell population could explain the lower level of pro-inflammatory cytokines in RPMI 10% FBS compared to serum-free media. However, no such heterogeneity was observed in serum-free media, as a result of the use of defined culture condition. In this context, it is important to account for the source of FBS. Since we have only tested the FBS from one provider, we cannot rule out the possibility that FBS used from a different source yields a different macrophage phenotype.

In conclusion, the data presented here highlight the influence of culture conditions on MDM phenotype and function, as a result of their high plasticity. It appears that generation of MDM in serum-free conditions leads to a homogeneous M1 or M2 population, suitable for functional studies investigating the biology of tumor promoting or tumoricidal macrophages *in vitro*. Analysis of tumor and tumor stroma derived factors in cancers of different etiology which contribute to either M1 or M2 macrophage education has the potential to reveal characteristics of tumors most likely to respond to macrophage targeting strategies such as antibodies neutralizing the function of CSF-1R [34].

## Supporting Information

Figure S1 **Effect of monocyte-derived macrophage supernatant on T47D cell line proliferation.** Evaluation was performed in the presence or the absence of neutralizing TNF-α antibodies, in (**A**) conditioned media from M1 or M2 MDM cultured in RPMI 10% FBS or XVivo 10, (**B**) conditioned media from activated M1 (act. M1), M2a and M2c MDM stimulated in XVivo 10. Identical y-axis scales were used for comparison sake. Data represent mean ± SEM of three independent experiments including each condition in triplicate. Statistical significance was determined using t-test pairwise comparison (** p<0.005).
(TIF)

Table S1 **Receptor expression level on monocytes and monocyte-derived macrophages.** (**A**) MDM were cultured for 6 days in RPMI 10% FBS or XVivo 10 media supplemented with either GM-CSF (M1) or M-CSF (M2); (**B**) MDM were cultured for 6 days in XVivo supplemented with the indicated cytokines. Data represent mean ± SEM of Mean Fluorescence Intensity (Geom. mean) of N donors. * indicates statistical significance between M1 and M2 MDM in Tukey-Kramer HSD test pairwise comparison.
(TIF)

Table S2 **Cytokine concentration in supernatant of monocyte-derived macrophages.** Monocytes were stimulated for 6 days with (**A**) GM-CSF (M1) or M-CSF (M2) in XVivo 10 or RPMI +10% FBS; or with (**B**) the indicated cytokines in XVivo 10. Data represent mean ± SEM of cytokine concentration in pg/ml of 8 donors in **A** and N donors in **B**. * indicates statistical significance between M1 and M2 MDM in Tukey-Kramer HSD test pairwise comparison.
(TIF)
