# Supplementary figures and images for: In Vitro Generation of Monocyte-Derived Macrophages under Serum-Free Conditions Improves Their Tumor Promoting Functions
Source: PLoS One. 2012 Aug 6;7(8):e42656. doi: 10.1371/journal.pone.0042656 (PMC3412794; doi:10.1371/journal.pone.0042656)

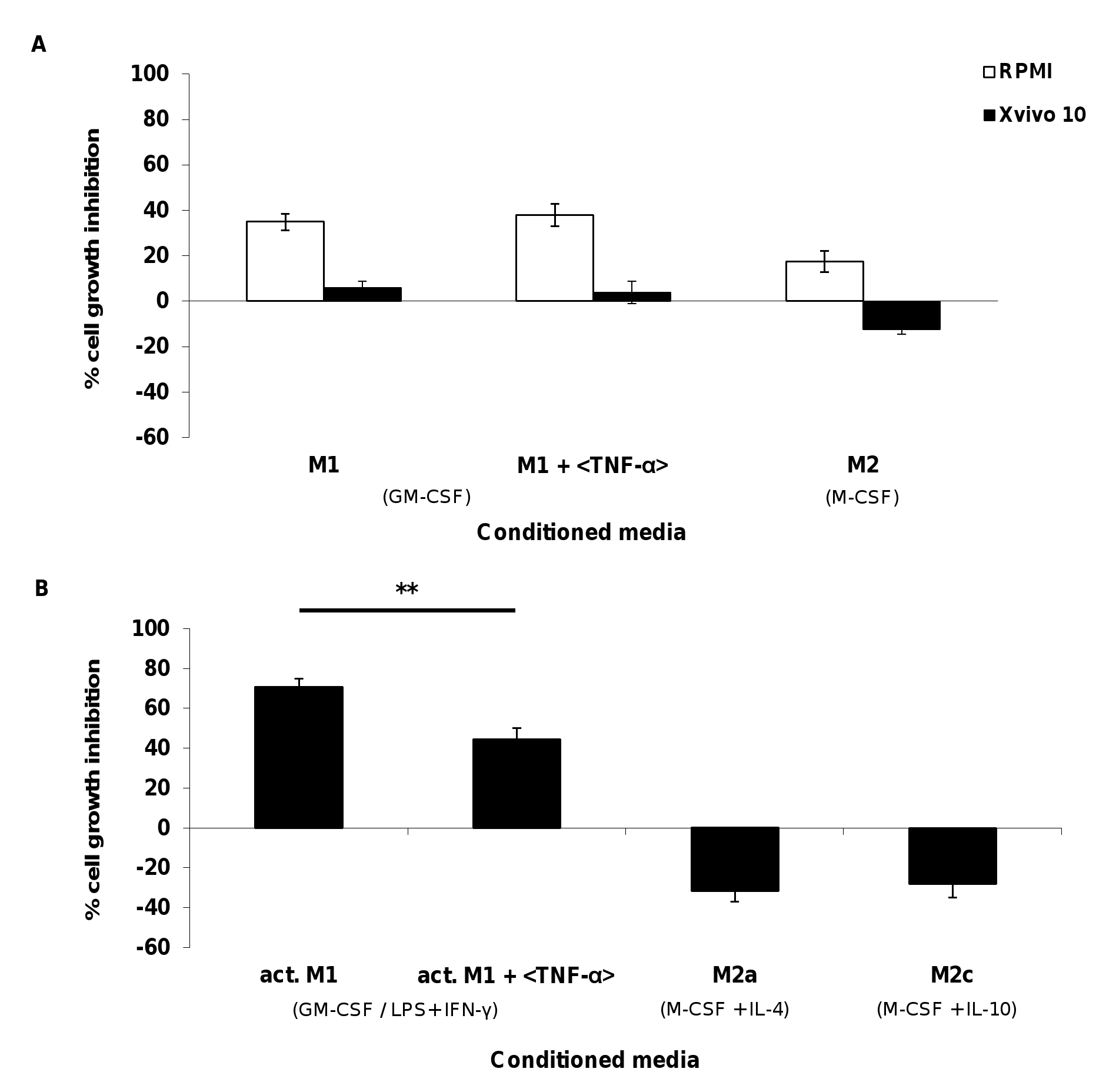

Supplement: Figure S1 — Effect of monocyte-derived macrophage supernatant on T47D cell line proliferation. Evaluation was performed in the presence or the absence of neutralizing TNF-α antibodies, in (A) conditioned media from M1 or M2 MDM cultured in RPMI 10% FBS or XVivo 10, (B) conditioned media from activated M1 (act. M1), M2a and M2c MDM stimulated in XVivo 10. Identical y-axis scales were used for comparison sake. Data represent mean ± SEM of three independent experiments including each condition in triplicate. Statistical significance was determined using t-test pairwise comparison (** p<0.005). (TIF) [file pone.0042656.s001.tif]

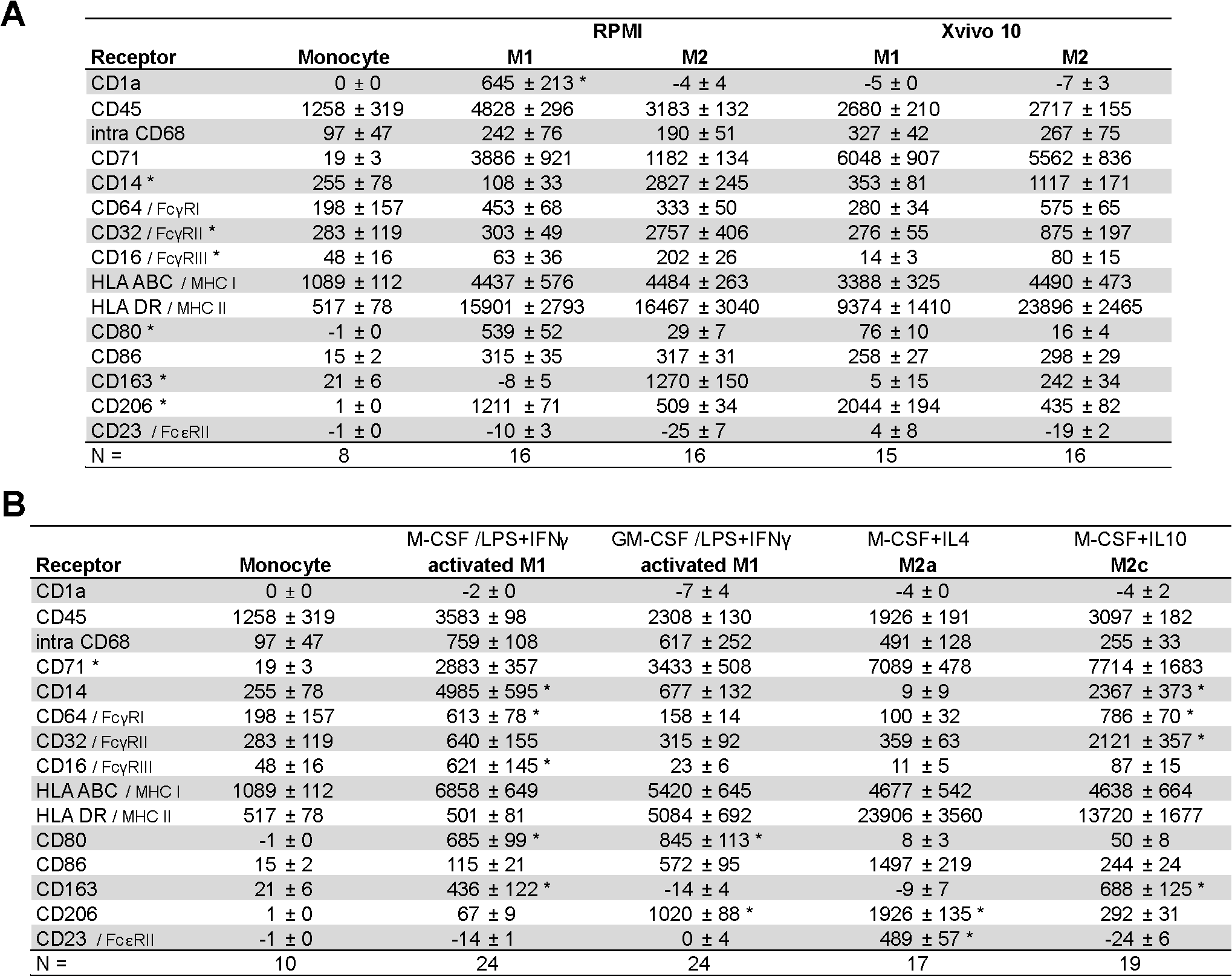

Supplement: Table S1 — Receptor expression level on monocytes and monocyte-derived macrophages. (A) MDM were cultured for 6 days in RPMI 10% FBS or XVivo 10 media supplemented with either GM-CSF (M1) or M-CSF (M2); (B) MDM were cultured for 6 days in XVivo supplemented with the indicated cytokines. Data represent mean ± SEM of Mean Fluorescence Intensity (Geom. mean) of N donors. * indicates statistical significance between M1 and M2 MDM in Tukey-Kramer HSD test pairwise comparison. (TIF) [file pone.0042656.s002.tif]

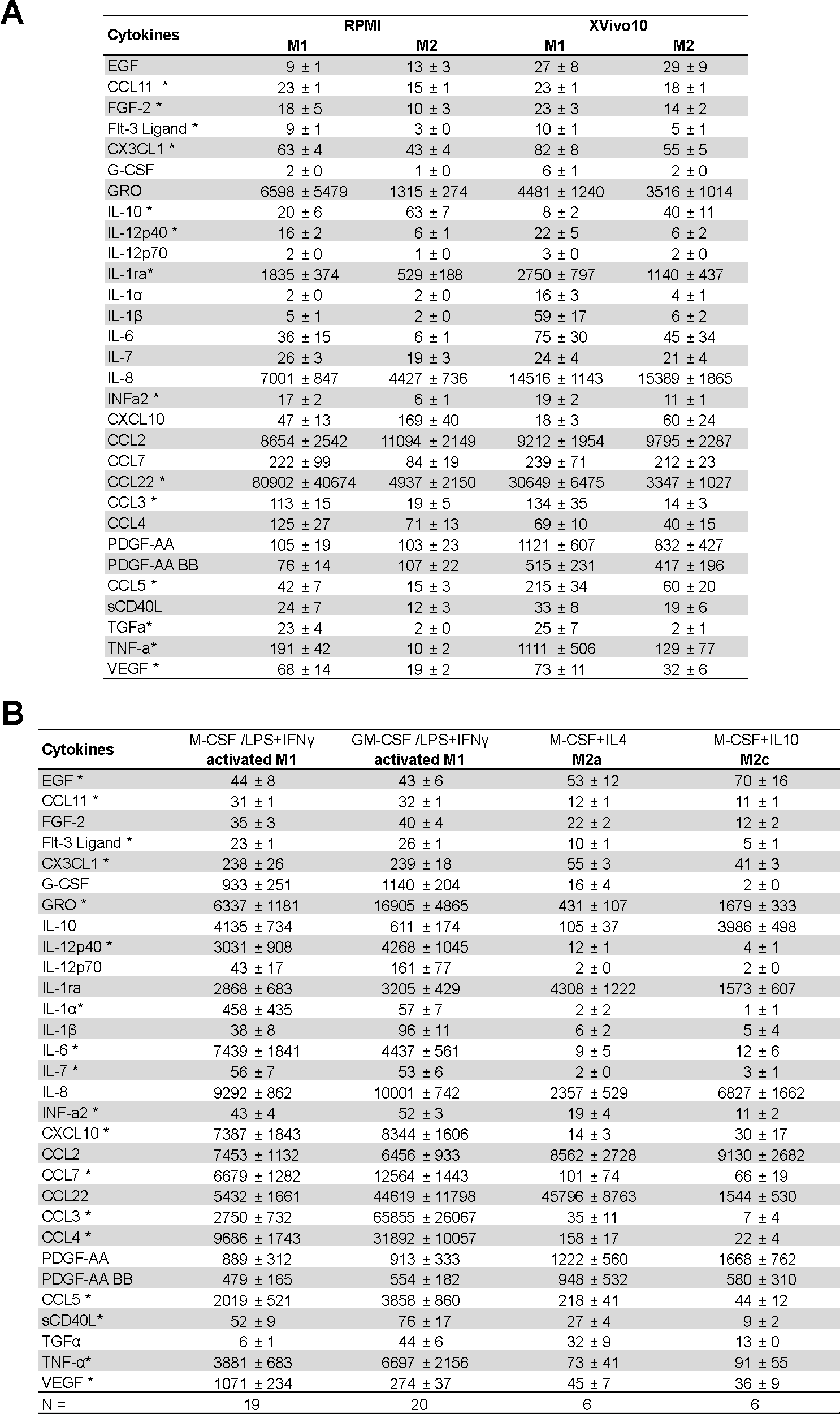

Supplement: Table S2 — Cytokine concentration in supernatant of monocyte-derived macrophages. Monocytes were stimulated for 6 days with (A) GM-CSF (M1) or M-CSF (M2) in XVivo 10 or RPMI +10% FBS; or with (B) the indicated cytokines in XVivo 10. Data represent mean ± SEM of cytokine concentration in pg/ml of 8 donors in A and N donors in B. * indicates statistical significance between M1 and M2 MDM in Tukey-Kramer HSD test pairwise comparison. (TIF) [file pone.0042656.s003.tif]
